# Retraction: Advancements in intrusion detection: A lightweight hybrid RNN-RF model

**DOI:** 10.1371/journal.pone.0319019

**Published:** 2025-02-06

**Authors:** The PLOS One Editors

The *PLOS One* Editors retract this article [1] due to concerns about peer review, reliability of the reported conclusions, and compliance with PLOS policies.

Specific concerns include:

- The article [1] reports conclusions about the accuracy of select Recurrent Neural Network classifiers that are not supported by the methodology or results.
- Elements within the article raised concerns about the article’s compliance with the PLOS Artificial Intelligence Tools and Technologies policy.

The corresponding author stated that no generative AI tools or automated text generation systems were used at any stage in the preparation of this article [1].

These concerns call into question the article’s validity and provenance. PLOS regrets that the issues were not identified prior to the article’s publication.

All authors did not agree with the retraction.
